# Distributed Optimal and Self-Tuning Filters Based on Compressed Data for Networked Stochastic Uncertain Systems with Deception Attacks

**DOI:** 10.3390/s23010335

**Published:** 2022-12-28

**Authors:** Yimin Ma, Shuli Sun

**Affiliations:** School of Electronic Engineering, Heilongjiang University, Harbin 150080, China

**Keywords:** multiplicative noise, weighted measurement fusion, unknown attack rate, identification, distributed self-tuning filter

## Abstract

In this study, distributed security estimation problems for networked stochastic uncertain systems subject to stochastic deception attacks are investigated. In sensor networks, the measurement data of sensor nodes may be attacked maliciously in the process of data exchange between sensors. When the attack rates and noise variances for the stochastic deception attack signals are known, many measurement data received from neighbour nodes are compressed by a weighted measurement fusion algorithm based on the least-squares method at each sensor node. A distributed optimal filter in the linear minimum variance criterion is presented based on compressed measurement data. It has the same estimation accuracy as and lower computational cost than that based on uncompressed measurement data. When the attack rates and noise variances of the stochastic deception attack signals are unknown, a correlation function method is employed to identify them. Then, a distributed self-tuning filter is obtained by substituting the identified results into the distributed optimal filtering algorithm. The convergence of the presented algorithms is analyzed. A simulation example verifies the effectiveness of the proposed algorithms.

## 1. Introduction

With the development of science and technology, networked systems or sensor networks [1] have been gradually applied to various key infrastructures. The networked systems introduce the network into a control system and realise data sharing among sensors, actuators, and controllers. The networked systems have the characteristics of low cost, simple maintenance, and flexibility. During data exchange between sensor nodes, data may be attacked maliciously by the networks. Methods to address the injected data in the state estimation are of importance. Therefore, the state estimation for systems with network attacks attracts considerable interest in the field of security estimation.

The types of network attacks mainly include deception, DoS, and replay attacks. The deception attack implies that an attacker injects false data into the network channels to affect the performance of the system [2]. The DoS attack implies that the attacker jams network channels to prohibit the transmission of data [3]. The replay attack is a special form of deception attack [4] in which an attacker puts captured historical data back into the channels. The current research on the three types of network attacks has attracted considerable attention. In [5], a deception attack model has been presented against state estimation in electric power grids. In [6], the distributed security filtering problem of wireless sensor networks under network deception attacks has been studied. By introducing an exponential function, a protector has been designed for each sensor node according to an innovation sequence, and an attack protection model is presented. In [7,8], the fusion estimation problem of deception attack signals has been studied for cyberphysical systems.

Under the network DoS attack, the event-triggered security estimation problems of sensor networks have been addressed in [9,10,11], whereas the distributed dimensional-reduction fusion estimators have been designed in [12,13]. In [14], the optimal DoS attack scheduling problem from the attacker’s perspective has been studied. The distributed detection for the DoS attack has also been developed in [15]. In [16], a model with a compensation has been developed to describe the replay attack, and then a recursive distributed estimator is devised in the LMV criterion. In [17], a distributed set membership filter is proposed for linear time-varying systems over sensor networks with limited bandwidths. The discrimination between the replay attack and sensor fault is investigated under an event-triggered transmission mechanism in [18]. In [19], the problem of detection and defence of replay attacks has been studied. In [20,21,22], the problem of security control and estimation under hybrid attacks has been also investigated.

In networked systems, there are many network-induced stochastic uncertainties in addition to network attacks, such as random delays, packet losses, and multiplicative noises, which affect the performance of the systems [1]. Stochastic uncertain systems have various applications; e.g., parameter errors in system models and fading channels in data transmissions can be depicted by multiplicative noises, and environmental disturbances can be often depicted by additive noises. For multisensor stochastic uncertain systems with random parameters, delays, and packet dropouts, a distributed fusion filter is presented in [23]. For multirate uncertain nonlinear systems with coloured measurement noises, a robust fusion algorithm has been designed in [24]. In the above literature, the statistical characteristics of noises are assumed to be known. Otherwise, adaptive or self-tuning estimation algorithms need to be designed. By using the correlation function method to identify the statistical characteristics of unknown noises, the distributed fusion self-tuning estimators have been proposed for multisensor systems with unknown noise variances in [25,26]. The distributed fusion self-tuning filters have also been developed for multisensor networked systems with unknown model parameters and data loss rates in [27,28,29]. For nonlinear systems, a fuzzy energy-to-peak filter [30] and distributed fusion filters [31] have also been studied. However, network attacks in networked systems are not involved in the above literature.

Compared to the centralised fusion estimation where the data of all nodes are transmitted to a fusion centre, the distributed fusion estimation based on the network topology has an advantage of resource sharing among sensor nodes. Each node acts as a local fusion centre. Each node can fuse the information from itself and its neighbours to improve the performance of the system. However, as a large amount of data from neighbour nodes is processed, an augmentation method will impose a costly computational overhead. To reduce the computational cost of the filter, the augmented measurement data received at each sensor node can be compressed to a dimensionality reduction measurement before being used for filtering. In addition, the data exchanged between sensor nodes may be subject to malicious attacks from the network. Therefore, it is vital to investigate the distributed security estimation problem of sensor networks based on data compression.

There have been few studies on the network security estimation of mixed uncertain systems subject to multiplicative noises, additive noises, including both state-dependent and noise-dependent multiplication and stochastic deception attacks. In this study, from the perspective of a defender, the distributed security estimation based on data compression is investigated for sensor networks. In contrast to [5,6,7,8,9,10,11,12], which only consider network attacks, and [23,24,25,26,27,28,29], which consider uncertainties of system model parameters and noise covariance, this study considers stochastic uncertainties of multiplicative noises, additive noises, and stochastic deception attacks. In contrast to the results on distributed estimation for multisensor systems in the above literature, where a large amount of data are not compressed and directly used by filters, data are first compressed and then used by filters in this study, which can reduce the computational burden of the filter. In contrast to the results on distributed estimation for systems with deception attacks in [32], where attack rates and noise variances of deception attack signals are assumed to be known, distributed self-tuning filters with unknown attack rates and noise variances of deception attack signals is designed in this study. However, attack rates and noise variances of deception attack signals are often unknown in practical applications.

The contributions of this paper are presented as follows.

(a) In the studied systems, mixed uncertainties of multiplicative noises, additive noises, and stochastic deception attacks are comprehensively considered, which can better reflect some practical systems.

(b) Under the known attack rates and noise variances of stochastic deception attack signals, a weighted measurement fusion algorithm in the least squares is used to compress the measurements of the sensor and its neighbours at each node, and then a distributed optimal filter is presented in the LMV. It has the same accuracy as that based on uncompressed data. Moreover, it has a lower computational cost than that based on uncompressed data.

(c) Under the unknown attack rates and noise variances of the stochastic deception attack signals, a correlation function method is employed to identify the attack rates and noise variances of attack signals at each node and then a distributed self-tuning filter is designed. The convergence of the distributed self-tuning filtering algorithm is analyzed; it converges to the distributed optimal filter if the identifications of attack rates and noise variances of attack signals are consistent.

The rest of this paper is organised as follows. The problem formulation is presented in Section 2. The distributed optimal filter is presented based on compressed data in Section 3. In Section 4, the distributed self-tuning filter based on compressed data is presented and its convergence is analyzed. An example is given in Section 5. Finally, conclusions are presented in Section 6.

Notations: $R^{n}$ represents the *n* -dimensional Euclidean space, $R^{n\times n}$ is the set of n×n real matrices, $A^{T}$ and $A^{-1}$ are the transpose and inverse of matrix *A*, respectively, $E\left\{•\right\}$ is the expectation, $\mathrm{Cov}\left\{•\right\}$ is the covariance, ρ(A) is the spectral radius of matrix *A*, $∥•∥$ is the Euclidean norm of a real vector or spectral norm of a real matrix, A⊗B is the Kronecker product of matrices *A* and *B*, and $\delta_{tk}$ is the Kronecker delta function.

## 2. Problem Formulation

Consider the multisensor, linear, time-invariant stochastic uncertain system,

(1) $$x\left(t+1\right)=\left(A+\sum_{l=1}^{q}\alpha_{l}\left(t\right)A_{l}\right)x\left(t\right)+\left(B+\sum_{l=1}^{q}\beta_{l}\left(t\right)B_{l}\right)\omega \left(t\right),$$

(2) $$y_{i}\left(t\right)=\left(C_{i}+\sum_{l=1}^{q}h_{il}\left(t\right)C_{il}\right)x\left(t\right)+v_{i}\left(t\right),\;\;\;\;\;\;\;i=1,2,\ldots ,L,$$

where $x\left(t\right)\in R^{n}$ is the system state, $y_{i}\left(t\right)\in R^{m_{i}}$ is the measurement of the *i*th sensor, $\alpha_{l}\left(t\right)\in R$, $\beta_{l}\left(t\right)\in R$, and $h_{il}\left(t\right)\in R$, l=1,2,…,q, are multiplicative noises to depict stochastic uncertainties of model parameters, where *q* is a positive integer, $\omega \left(t\right)\in R^{r}$ is the process noise, $v_{i}\left(t\right)\in R^{m_{i}}$ is the measurement noise, and *A*, *B*, $C_{i}$, $A_{l}$, $B_{l}$ and $C_{il}$ are constant matrices with appropriate dimensions. The subscript *i* corresponds to the *i*th sensor, and *L* is the number of sensors.

**Assumption** **1.** *Multiplicative noises $\alpha_{l}\left(t\right)\in R$, $\beta_{l}\left(t\right)\in R$, and $h_{il}\left(t\right)\in R$ are uncorrelated white noises with zero mean and covariance $Q_{\alpha_{l}}$, $Q_{\beta_{l}}$, and $Q_{h_{il}}$, respectively; process noise $\omega \left(t\right)\in R^{r}$ and measurement noise $v_{i}\left(t\right)$ are uncorrelated white noises with zero mean and covariance $Q_{\omega }$ and $Q_{v_{i}}$. Moreover, multiplicative noises $\alpha_{l}\left(t\right)$, $\beta_{l}\left(t\right)$, and $h_{il}\left(t\right)$ are uncorrelated with additive noises ω(t) and $v_{i}\left(t\right)$.*

**Assumption** **2.** *The initial state value x(0) is uncorrelated with ω(t), $v_{i}\left(t\right)$, $\alpha_{l}\left(t\right)$, $\beta_{l}\left(t\right)$, and $h_{il}\left(t\right)$, with the mean and covariance as*

(3) $$E\left\{x(0)\right\}=\mu_{0},E\left\{\left[x\left(0\right)-\mu_{0}\right]{\left[x\left(0\right)-\mu_{0}\right]}^{T}\right\}=P_{0}.$$

**Assumption** **3.** *A is a stable matrix, and $\rho (A⊗A+\sum_{l=1}^{q}Q_{\alpha_{l}}A_{l}⊗A_{l})<1$.*

Assumption 1 describes the statistical characteristics of noises. Assumption 2 provides the statistical characteristics of the initial state. They are applicable to state estimation problems in general [23,33]. Assumption 3 implies that the studied systems are stable in the mean square sense, which guarantees the existence of the state second moment in the later text [34].

We consider a sensor network consisting of *L* sensor nodes. Its topology is described by a graph G=(V,E), where V={1,2,…,L} is the set of sensor nodes, and E={(i,j):i,j∈V}⊂V×V is the edge set formed by the interactive connections between nodes. We denote the set of neighbour nodes of sensor *i* by $N_{i}=\left\{j\in V:\left(j,i\right)\in \;E\right\}$, where (j,i)∈E indicates that the sensor *i* can receive the data transmitted by its neighbour node *j*. We denote the number of neighbour nodes of sensor *i* as $d_{i}$.

In the process of data exchange between nodes, the measurement data may be attacked maliciously by the network. We consider the following form of network deception attack signals when the sensor *i* transmits its measurement data through the network,

(4) $${\vec{y}}_{i}\left(t\right)=-y_{i}\left(t\right)+\sigma_{i}\left(t\right),\;\;\;\;\;\;\;i=1,2,\ldots ,L,$$

where $\sigma_{i}\left(t\right)$ is a white noise with zero-mean and variance $Q_{\sigma_{i}}$, independent of other random variables.

Considering the limited energy of the attacker and limited network source, the attack does not always exist and may occur randomly. If we assume that the attack signal satisfies Bernoulli distribution in the network, the measurement data of the attacked sensor node *i* satisfies the following equation,

(5) $${\bar{y}}_{i}\left(t\right)=y_{i}\left(t\right)+\gamma_{i}\left(t\right){\vec{y}}_{i}\left(t\right)\;,$$

where $\gamma_{i}\left(t\right)$ is a Bernoulli random variable with the following known statistical characteristics $E\left\{\gamma_{i}\left(t\right)=1\right\}={\bar{\gamma }}_{i}$, $E\left\{\gamma_{i}\left(t\right)=0\right\}=1-{\bar{\gamma }}_{i}$, $\mathrm{Cov}\left\{\gamma_{i}\left(t\right)\right\}={\bar{\gamma }}_{i}\left(1-{\bar{\gamma }}_{i}\right)$, $0\leq {\bar{\gamma }}_{i}\leq 1$. In the model (5), if $\gamma_{i}\left(t\right)=0$ implies the absence of an attack, $\gamma_{i}\left(t\right)=1$ implies a complete attack. Thus, model (5) is more general.

The purpose of this study is to devise a distributed optimal filter in the LMV sense under the known attack rates ${\bar{\gamma }}_{i_{k}}$ and noise variances $Q_{\sigma_{i_{k}}}$, $i_{k}\in N_{i}$ of the deception attack signals, and distributed self-tuning filter under the unknown attack rates ${\bar{\gamma }}_{i_{k}}$ and noise variances $Q_{\sigma_{i_{k}}}$, $i_{k}\in N_{i}$ of the deception attack signals at each sensor node *i*, based on its measurement data $y_{i}\left(t\right)$ and measurement data ${\bar{y}}_{i_{k}}\left(t\right)$, $k=1,2,...,d_{i}$ received from its neighbour nodes $i_{k}\in N_{i}$.

**Remark** **1.** *The studied systems contain uncertainties due to multiplicative and additive noises. Multiplicative noises can be used to describe parameter errors in system modelling and signal transmission fading. Additive noises can be used to describe the background environmental disturbances of the systems.*

## 3. Distributed Optimal Filter

Before presenting a distributed self-tuning filter, a distributed optimal filter is first presented in this section. By compressing measurement data of sensor itself and neighbor nodes, a distributed optimal filter in the LMV criterion is devised under the condition that the attack rates and noise variances of the deception attack signals are known.

### 3.1. Model Transformation

At sensor node *i*, a distributed optimal filter is devised based on the measurements $y_{i}\left(t\right)$ and ${\bar{y}}_{i_{k}}\left(t\right)$ of it and its neighbour nodes $i_{k}\in N_{i}$. However, the received measurement data ${\bar{y}}_{i_{k}}\left(t\right)$ from its neighbour nodes $i_{k}\in N_{i}$ may be subject to deception attacks. Systems (1) and (2) is then transformed as follows:

(6) $$x\left(t+1\right)=Ax\left(t\right)+\underline{\omega }\left(t\right)$$

(7) $$y_{i}\left(t\right)=C_{i}x\left(t\right)+{\underline{v}}_{i}\left(t\right)$$

(8) $${\bar{y}}_{i_{k}}\left(t\right)={\bar{C}}_{i_{k}}x\left(t\right)+{\bar{v}}_{i_{k}}\left(t\right),\;\;\;\;\;i_{k}\in N_{i}$$

where

(9) $${\bar{C}}_{i_{k}}=\left(1-{\bar{\gamma }}_{i_{k}}\right)C_{i_{k}}$$

(10) $$\underline{\omega }\left(t\right)=\sum_{l=1}^{q}\alpha_{l}\left(t\right)A_{l}x\left(t\right)+B\omega \left(t\right)+\sum_{l=1}^{q}\beta_{l}\left(t\right)B_{l}\omega \left(t\right)$$

(11) $${\underline{v}}_{i}\left(t\right)=v_{i}\left(t\right)+\sum_{l=1}^{q}h_{il}\left(t\right)C_{il}x\left(t\right)$$

(12) $${\bar{v}}_{i_{k}}\left(t\right)=\left[{\bar{\gamma }}_{i_{k}}-\gamma_{i_{k}}\left(t\right)\right]C_{i_{k}}x\left(t\right)+\left[1-\gamma_{i_{k}}\left(t\right)\right]\sum_{l=1}^{q}h_{i_{k}l}\left(t\right)C_{i_{k}l}x\left(t\right)+\gamma_{i_{k}}\left(t\right)\sigma_{i_{k}}\left(t\right)+\left[1-\gamma_{i_{k}}\left(t\right)\right]v_{i_{k}}\left(t\right).$$

${\bar{y}}_{i_{k}}\left(t\right)$, $i_{k}\in N_{i},k=1,2,\cdots ,d_{i}$ are the measurements of the neighbour nodes of sensor node *i*. $\underline{\omega }\left(t\right)$ is the new process noise, ${\underline{v}}_{i}\left(t\right)$ is the new measurement noise of the transformed systems (6) and (7), and ${\bar{v}}_{i_{k}}\left(t\right)$, $i_{k}\in N_{i}$ are the measurement noises of the neighbour nodes of sensor node *i*. $\underline{\omega }\left(t\right)$, ${\underline{v}}_{i}\left(t\right)$, and ${\bar{v}}_{i_{k}}\left(t\right)$ are still white noises of zero-mean and covariance matrices $Q_{\underline{\omega }}\left(t\right)=E\left\{\underline{\omega }\left(t\right)\underline{\omega }{}^{T}\left(t\right)\right\}$, $Q_{{\underline{v}}_{i}}\left(t\right)=E\left\{{\underline{v}}_{i}\left(t\right){\underline{v}}_{i}^{T}\left(t\right)\right\}$, $Q_{{\bar{v}}_{i_{k}}}\left(t\right)=E\left\{{\bar{v}}_{i_{k}}\left(t\right){\bar{v}}_{i_{k}}^{T}\left(t\right)\right\}$:

(13) $$Q_{\underline{\omega }}\left(t\right)=\sum_{l=1}^{q}Q_{\alpha_{l}}A_{l}X\left(t\right)A_{l}^{T}+BQ_{\omega }B^{T}+\sum_{l=1}^{q}Q_{\beta_{l}}B_{l}Q_{\omega }B_{l}^{T}$$

(14) $$Q_{{\underline{v}}_{i}}\left(t\right)=Q_{v_{i}}+\sum_{l=1}^{q}Q_{h_{il}}C_{il}X\left(t\right)C_{il}^{T}$$

(15) $$Q_{{\bar{v}}_{i_{k}}}\left(t\right)={\bar{\gamma }}_{i_{k}}\left(1-{\bar{\gamma }}_{i_{k}}\right)C_{i_{k}}X\left(t\right)C_{i_{k}}^{T}+\left(1-{\bar{\gamma }}_{i_{k}}\right)\sum_{l=1}^{q}Q_{h_{i_{k}l}}C_{i_{k}l}X\left(t\right)C_{i_{k}l}^{T}+{\bar{\gamma }}_{i_{k}}Q_{\sigma_{i_{k}}}+(1-{\bar{\gamma }}_{i_{k}})Q_{v_{i_{k}}}.$$

According to (1), the state second moment $X\left(t\right)=E\left\{x\left(t\right)x^{T}\left(t\right)\right\}$ can be recursively calculated as

(16) $$X\left(t+1\right)=AX\left(t\right)A^{T}+\sum_{l=1}^{q}Q_{\alpha_{l}}A_{l}X\left(t\right)A_{l}^{T}+BQ_{\omega }B^{T}+\sum_{l=1}^{q}Q_{\beta_{l}}B_{l}Q_{\omega }B_{l}^{T},$$

with an initial of value $X\left(0\right)=\mu_{0}\mu_{0}^{T}+P_{0}$.

Under Assumption 3, the state second moment X(t) is bounded [34]. Thus, $Q_{\underline{\omega }}\left(t\right)$ in (13), $Q_{{\underline{v}}_{i}}\left(t\right)$ in (14), and $Q_{{\bar{v}}_{i_{k}}}\left(t\right)$ in (15) are also bounded, which is necessary for the filter design.

Based on the measurements (7) and (8), each node augments its measurement $y_{i}\left(t\right)$ and receives measurements ${\bar{y}}_{i_{k}}\left(t\right)$ of its neighbour nodes. The augmented measurement equation is

(17) $$Y_{i}^{\left(a\right)}\left(t\right)=C_{i}^{\left(a\right)}x\left(t\right)+V_{i}^{\left(a\right)}\left(t\right),$$

where $Y_{i}^{\left(a\right)}\left(t\right)={\left[y_{i}^{T}\left(t\right),{\bar{y}}_{i_{1}}^{T}\left(t\right),\cdots ,{\bar{y}}_{i_{d_{i}}}^{T}\left(t\right)\right]}^{T}$, $C_{i}^{\left(a\right)}={\left[C_{i}^{T},{\bar{C}}_{i_{1}}^{T},\cdots ,{\bar{C}}_{i_{d_{i}}}^{T}\right]}^{T}$, $V_{i}^{\left(a\right)}\left(t\right)=\left[{\underline{v}}_{i}^{T}\left(t\right)\right.$, ${\left.\;{\bar{V}}_{i}^{T}\left(t\right)\right]}^{T}$ and ${\bar{V}}_{i}\left(t\right)=\left[{\bar{v}}_{i_{1}}^{T}\left(t\right),\cdots ,\;\right.\left.\;{\bar{v}}_{i_{d_{i}}}^{T}\left(t\right)\right]$. The superscript (a) denotes the augmentation.

The statistical characteristic of the noise $V_{i}^{\left(a\right)}\left(t\right)$ is

$$Q_{V_{i}^{\left(a\right)}}(t)=E\left\{V_{i}^{\left(a\right)}\left(t\right){\left(V_{i}^{\left(a\right)}\left(t\right)\right)}^{T}\right\}=\mathrm{diag}\left[Q_{{\underline{v}}_{i}}\left(t\right),Q_{{\bar{V}}_{i}}^{}\left(t\right)\right],$$

where $Q_{{\bar{V}}_{i}}^{}\left(t\right)=E\left\{{\bar{V}}_{i}\left(t\right){\bar{V}}_{i}^{T}\left(t\right)\right\}=\mathrm{diag}\left[Q_{{\bar{v}}_{i_{1}}}\left(t\right),\;\;Q_{{\bar{v}}_{i_{2}}}\left(t\right),\cdots \;\;,\;Q_{{\bar{v}}_{i_{d_{i}}}}\left(t\right)\right]$.

For the state Equation (6) and augmented measurement (17), the standard Kalman filtering algorithm [35] can be applied to obtain the distributed filter at each sensor node. However, the distributed filter based on the augmented measurement (17) has a heavy computational cost due to the high dimension of the augmented measurement, where the gain matrix requires the inverse of a high-dimensional matrix. To overcome this shortcoming, the augmented high-dimensional measurement can be compressed to a low-dimensional measurement, and then the filter is designed based on the compressed data, reducing the computational burden.

### 3.2. DOFCD

An augmented measurement is compressed to a dimensionality reduction measurement using a weighted least-squares algorithm [36] in this subsection.

For the augmented measurement Equation (17), if $\mathrm{rank}\left\{C_{i}^{\left(a\right)}\right\}=r_{i}⩽\min\left\{n,{\bar{m}}_{i}\right\}$, where ${\bar{m}}_{i}=m_{i}+\sum_{k=1}^{d_{i}}m_{i_{k}}$, there is a full rank decomposition:

(18) $$C_{i}^{\left(a\right)}=F_{i}^{\left(c\right)}C_{i}^{\left(c\right)},$$

where $F_{i}^{\left(c\right)}\in R^{{\bar{m}}_{i}\times r_{i}}$ is a full column rank matrix and $C_{i}^{\left(c\right)}\in R^{r_{i}\times n}$ is a full row rank matrix. Let

(19) $$F_{i}^{\left(c\right)}={\left[F_{i}^{T},F_{i_{1}}^{T},\cdots ,F_{i_{d_{i}}}^{T}\right]}^{T}.$$

The augmented measurement (17) can be rewritten as

(20) $$Y_{i}^{\left(a\right)}\left(t\right)=F_{i}^{\left(c\right)}C_{i}^{\left(c\right)}x\left(t\right)+V_{i}^{\left(a\right)}\left(t\right).$$

By applying the weighted least-squares algorithm to compress the measurement, we obtain

$${\left[F_{i}^{T}Q_{{\underline{v}}_{i}}^{-1}\left(t\right)F_{i}+\sum_{k=1}^{d_{i}}F_{i_{k}}^{T}Q_{{\bar{v}}_{i_{k}}}^{-1}\left(t\right)F_{i_{k}}\right]}^{-1}\left[F_{i}^{T}Q_{{\underline{v}}_{i}}^{-1}\left(t\right)y_{i}\left(t\right)+\sum_{k=1}^{d_{i}}F_{i_{k}}^{T}Q_{{\bar{v}}_{i_{k}}}^{-1}\left(t\right){\bar{y}}_{i_{k}}\left(t\right)\right]$$

(21) $$=C_{i}^{\left(c\right)}x\left(t\right)+[F_{i}^{T}Q_{{\underline{v}}_{i}}^{-1}\left(t\right)F_{i}+\sum_{k=1}^{d_{i}}F_{i_{k}}^{T}Q_{{\bar{v}}_{i_{k}}}^{-1}\left(t\right)F_{i_{k}}{]}^{-1}\left[F_{i}^{T}Q_{{\underline{v}}_{i}}^{-1}\left(t\right){\underline{v}}_{i}\left(t\right)+\sum_{k=1}^{d_{i}}F_{i_{k}}^{T}Q_{{\bar{v}}_{i_{k}}}^{-1}\left(t\right){\bar{v}}_{i_{k}}\left(t\right)\right]$$

Let $Y_{i}^{\left(c\right)}\left(t\right)={\left[F_{i}^{T}Q_{{\underline{v}}_{i}}^{-1}\left(t\right)F_{i}+\sum_{k=1}^{d_{i}}F_{i_{k}}^{T}Q_{{\bar{v}}_{i_{k}}}^{-1}\left(t\right)F_{i_{k}}\right]}^{-1}\left[F_{i}^{T}Q_{{\underline{v}}_{i}}^{-1}\left(t\right)y_{i}\left(t\right)+\sum_{k=1}^{d_{i}}F_{i_{k}}^{T}Q_{{\bar{v}}_{i_{k}}}^{-1}\left(t\right){\bar{y}}_{i_{k}}\left(t\right)\right]$ and $V_{i}^{\left(c\right)}\left(t\right)=[F_{i}^{T}Q_{{\underline{v}}_{i}}^{-1}\left(t\right)F_{i}+\sum_{k=1}^{d_{i}}F_{i_{k}}^{T}Q_{{\bar{v}}_{i_{k}}}^{-1}\left(t\right)F_{i_{k}}{]}^{-1}\left[F_{i}^{T}Q_{{\underline{v}}_{i}}^{-1}\left(t\right){\underline{v}}_{i}\left(t\right)+\sum_{k=1}^{d_{i}}F_{i_{k}}^{T}Q_{{\bar{v}}_{i_{k}}}^{-1}\left(t\right){\bar{v}}_{i_{k}}\left(t\right)\right]$ and the following compressed measurement equation is obtained:

(22) $$Y_{i}^{\left(c\right)}\left(t\right)=C_{i}^{\left(c\right)}x\left(t\right)+V_{i}^{\left(c\right)}\left(t\right),$$

where the superscript (c) denotes compression. The new measurement $Y_{i}^{\left(c\right)}\left(t\right)$ has a reduced dimension $r_{i}⩽\min\left\{n,{\bar{m}}_{i}\right\}$. $V_{i}^{\left(c\right)}\left(t\right)$ has a variance matrix of

(23) $$Q_{V_{i}^{\left(c\right)}}^{}\left(t\right)={\left[F_{i}^{T}Q_{{\underline{v}}_{i}}^{-1}\left(t\right)F_{i}+\sum_{k=1}^{d_{i}}F_{i_{k}}^{T}Q_{{\bar{v}}_{i_{k}}}^{-1}\left(t\right)F_{i_{k}}\right]}^{-1}.$$

For state Equation (6) and compressed low-dimensional measurement Equation (22), the following filter is obtained by applying the standard Kalman filtering algorithm [35].

**Theorem** **1.** *For systems (6) and (22), the DOFCD in the LMV criterion is calculated as follows*

(24) $${\hat{x}}_{i}^{\left(c\right)}\left(t+1|t+1\right)={\hat{x}}_{i}^{\left(c\right)}\left(t+1|t\right)+K_{i}^{\left(c\right)}\left(t+1\right)\left(Y_{i}^{\left(c\right)}\left(t+1\right)-C_{i}^{\left(c\right)}{\hat{x}}_{i}^{\left(c\right)}\left(t+1|t\right)\right)$$

(25) $${\hat{x}}_{i}^{\left(c\right)}\left(t+1|t\right)=A{\hat{x}}_{i}^{\left(c\right)}\left(t|t\right)$$

(26) $$K_{fi}^{\left(c\right)}\left(t+1\right)=P_{i}^{\left(c\right)}\left(t+1|t\right)C_{i}^{\left(c\right)T}{\left[C_{i}^{\left(c\right)}P_{i}^{\left(c\right)}\left(t+1|t\right)C_{i}^{\left(c\right)T}+Q_{V_{i}^{\left(c\right)}}\left(t+1\right)\right]}^{-1}$$

(27) $$P_{i}^{\left(c\right)}\left(t+1|t\right)=AP_{i}^{\left(c\right)}\left(t|t\right)A^{T}+Q_{\underline{\omega }}\left(t\right)$$

(28) $$P_{i}^{\left(c\right)}\left(t+1|t+1\right)=\left[I_{n}-K_{i}^{\left(c\right)}\left(t+1\right)C_{i}^{\left(c\right)}\right]P_{i}^{\left(c\right)}\left(t+1|t\right),$$

*where ${\hat{x}}_{i}^{\left(c\right)}\left(t+1|t+1\right)$ and ${\hat{x}}_{i}^{\left(c\right)}\left(t+1|t\right)$ are the filtering and prediction estimates of sensor node i based on the compressed measurement, respectively, $K_{fi}^{\left(c\right)}\left(t+1\right)$ is the corresponding filtering gain matrix, and $P_{i}^{\left(c\right)}\left(t+1|t+1\right)$ and $P_{i}^{\left(c\right)}\left(t+1|t\right)$ are the filtering error variance and prediction error variance, respectively. The initial values are ${\hat{x}}_{i}^{\left(c\right)}\left(0|0\right)=\mu_{0}$ and $P_{i}^{\left(c\right)}\left(0|0\right)=P_{0}$.*

**Remark** **2.** *Compared to the distributed optimal filter based on the augmented measurement with the computational complexity $O({{\bar{m}}_{i}}^{3}+n^{3})$, the distributed optimal filter based on the compressed measurement in Theorem 1 with the computational complexity $O(n^{3})$ has a lower computational cost. In particular, when there are a large number of neighbour sensor nodes, i.e., $n\ll {\bar{m}}_{i}$, the distributed filter based on compressed data proposed in Theorem 1 significantly reduce the computational cost. Moreover, they have the same estimation accuracy [36].*

## 4. Distributed Self-Tuning Filter

In the preceding section, the distributed optimal filter has been designed under the assumption of known attack rates and noise variances of the stochastic deception attack signals. However, the attack rates and noise variances of the stochastic deception attack signals are usually unknown in practical systems. The distributed optimal filter proposed in Section 3 cannot be applied. In this section, we devise a distributed self-tuning filtering algorithm for the case when the attack rates and noise variances of the stochastic deception attack signals are unknown.

### 4.1. Identification of Attack Rates and Noise Variances of Deception Attack Signals

If the attack rates ${\bar{\gamma }}_{i_{k}}$ and noise variances of the stochastic deception attack signals $Q_{\sigma_{i_{k}}}$, $i_{k}\in N_{i}$ are unknown, the unknown attack rates ${\bar{\gamma }}_{i_{k}}$ and noise variances $Q_{\sigma_{i_{k}}}$, $i_{k}\in N_{i}$ must be identified first to apply the distributed optimal filtering algorithm in Theorem 1 for state estimation. The real-time identified attack rates ${\hat{\bar{\gamma }}}_{i_{k}}\left(t\right)$ and noise variances ${\hat{Q}}_{\sigma_{i_{k}}}(t)$ are then substituted into Theorem 1 to obtain a distributed self-tuning filter.

The attack rates ${\bar{\gamma }}_{i_{k}}$ and noise variances $Q_{\sigma_{i_{k}}}$, $i_{k}\in N_{i}$ are identified by a correlation function method. By using (2), (4), and (5), we obtain

(29) $${\bar{y}}_{i_{k}}\left(t\right)=\left[1-\gamma_{i_{k}}\left(t\right)\right]C_{i_{k}}x\left(t\right)+\left[1-\gamma_{i_{k}}\left(t\right)\right]\sum_{l=1}^{q}h_{i_{k}l}\left(t\right)C_{i_{k}l}x\left(t\right)+\left[1-\gamma_{i_{k}}\left(t\right)\right]v_{i_{k}}\left(t\right)+\gamma_{i_{k}}\left(t\right).\sigma_{i_{k}}\left(t\right).$$

The zero-order correlation function of the measurement is calculated as

$$R_{i_{k}}\left(t,0\right)=E\left[{\bar{y}}_{i_{k}}\left(t\right){\bar{y}}_{i_{k}}^{T}\left(t\right)\right]$$

$$=\;E\{{\left(1-\gamma_{i_{k}}\left(t\right)\right)}^{2}C_{i_{k}}x\left(t\right)x^{T}\left(t\right)C_{i_{k}}^{T}\}$$

$$+E\{{\left(1-\gamma_{i_{k}}\left(t\right)\right)}^{2}\sum_{l=1}^{q}h_{i_{k}l}\left(t\right)C_{i_{k}l}x\left(t\right)x^{T}\left(t\right)\sum_{l=1}^{q}C_{i_{k}l}^{T}h_{i_{k}l}^{T}\left(t\right)\}$$

$$+E\left\{{\left(1-\gamma_{i_{k}}\left(t\right)\right)}^{2}v_{i_{k}}\left(t\right)v_{i_{k}}^{T}\left(t\right)\right\}+E\{[\gamma_{i_{k}}\left(t\right){]}^{2}\sigma_{i_{k}}\left(t\right)\sigma_{i_{k}}^{T}\left(t\right)\}$$

(30) $$=\left(1-{\bar{\gamma }}_{i_{k}}\right)\left[C_{i_{k}}X\left(t\right)C_{i_{k}}^{T}+\sum_{l=1}^{q}Q_{h_{i_{k}l}}C_{i_{k}l}X\left(t\right)C_{i_{k}l}^{T}+Q_{v_{i_{k}}}\right]+{\bar{\gamma }}_{i_{k}}Q_{\sigma_{i_{k}}}.$$

The first-order correlation function of the measurement is calculated as

$$R_{i_{k}}\left(t,1\right)=E\left[{\bar{y}}_{i_{k}}\left(t\right){\bar{y}}_{i_{k}}^{T}\left(t-1\right)\right]$$

$$=\;E\{(1-\gamma_{i_{k}}\left(t\right)\left(1-\gamma_{i_{k}}\left(t-1\right)\right)C_{i_{k}}Ax\left(t-1\right)x^{T}\left(t-1\right)C_{i_{k}}^{T}\}$$

(31) $$={\left(1-{\bar{\gamma }}_{i_{k}}\right)}^{2}C_{i_{k}}AX\left(t-1\right)C_{i_{k}}^{T},$$

which uses the results $E\left\{{\left(1-\gamma_{i_{k}}\left(t\right)\right)}^{2}\right\}=1-{\bar{\gamma }}_{i_{k}}$. $E\left\{{\left[\gamma_{i_{k}}\left(t\right)\right]}^{2}\right\}={\bar{\gamma }}_{i_{k}}$, and $E\left\{\left(1-\gamma_{i_{k}}\left(t\right)\right)\left(1-\gamma_{i_{k}}\left(t-1\right)\right)\right\}={\left(1-{\bar{\gamma }}_{i_{k}}\right)}^{2}$.

Thus, according to (30) and (31),

(32) $${\hat{\bar{\gamma }}}_{i_{k}}\left(t\right)=1-{\left(\mathrm{tr}R_{i_{k}}\left(t,1\right)/\mathrm{tr}\left(C_{i_{k}}AX\left(t-1\right)C_{i_{k}}^{T}\right)\right)}^{1/2}$$

(33) $${\hat{Q}}_{\sigma_{i_{k}}}(t)={\hat{\bar{\gamma }}}_{i_{k}}^{-1}\left(t\right)\left\{R_{i_{k}}\left(t,0\right)-\left(1-{\hat{\bar{\gamma }}}_{i_{k}}\left(t\right)\right)\left[C_{i_{k}}X\left(t\right)C_{i_{k}}^{T}+\sum_{l=1}^{q}Q_{h_{i_{k}l}}C_{i_{k}l}X\left(t\right)C_{i_{k}l}^{T}+Q_{v_{i_{k}}}\right]\right\}.$$

The correlation functions of the measurement $R_{i_{k}}\left(t,0\right)=E\left[{\bar{y}}_{i_{k}}\left(t\right){\bar{y}}_{i_{k}}^{T}\left(t\right)\right]$ and $R_{i_{k}}\left(t,1\right)=E\left[{\bar{y}}_{i_{k}}\left(t\right){\bar{y}}_{i_{k}}^{T}\left(t-1\right)\right]$ can be calculated approximately by the sampled correlation functions [25],

(34) $${\hat{R}}_{i_{k}}\left(t,0\right)=\frac{1}{t}\sum_{k=1}^{t}{\bar{y}}_{i_{k}}\left(k\right){\bar{y}}_{i_{k}}^{T}\left(k\right)$$

(35) $${\hat{R}}_{i_{k}}\left(t,1\right)=\frac{1}{t}\sum_{k=1}^{t}{\bar{y}}_{i_{k}}\left(k\right){\bar{y}}_{i_{k}}^{T}\left(k-1\right),$$

which can be recursively calculated by

(36) $${\hat{R}}_{i_{k}}\left(t,0\right)={\hat{R}}_{i_{k}}\left(t-1,0\right)+\frac{1}{t}\left[{\bar{y}}_{i_{k}}\left(t\right){\bar{y}}_{i_{k}}^{T}\left(t\right)-{\hat{R}}_{i_{k}}\left(t-1,0\right)\right]$$

(37) $${\hat{R}}_{i_{k}}\left(t,1\right)={\hat{R}}_{i_{k}}\left(t-1,1\right)+\frac{1}{t}\left[{\bar{y}}_{i_{k}}\left(t\right){\bar{y}}_{i_{k}}^{T}\left(t-1\right)-{\hat{R}}_{i_{k}}\left(t-1,1\right)\right].$$

By replacing $R_{i_{k}}\left(t,0\right)$ in (30) by ${\hat{R}}_{i_{k}}\left(t,0\right)$, and $R_{i_{k}}\left(t,1\right)$ in (31) by ${\hat{R}}_{i_{k}}\left(t,1\right)$, we can obtain the identified value ${\hat{\bar{\gamma }}}_{i_{k}}(t)$ of ${\bar{\gamma }}_{i_{k}}$, and ${\hat{Q}}_{\sigma_{i_{k}}}(t)$ of $Q_{\sigma_{i_{k}}}$. As the sampled correlation function converges to the true correlation function [25], i.e., ${\hat{R}}_{i_{k}}\left(t,0\right)\to R_{i_{k}}\left(t,0\right)$, ${\hat{R}}_{i_{k}}\left(t,1\right)\to R_{i_{k}}\left(t,1\right),t\to \infty$, the identified values ${\hat{\bar{\gamma }}}_{i_{k}}(t)$ and ${\hat{Q}}_{\sigma_{i_{k}}}(t)$ are consistent, i.e.,

(38) $${\hat{\bar{\gamma }}}_{i_{k}}(t)\to {\bar{\gamma }}_{i_{k}},t\to \infty$$

(39) $${\hat{Q}}_{\sigma_{i_{k}}}(t)\to Q_{\sigma_{i_{k}}},t\to \infty .$$

**Remark** **3.** *Under no network attacks, the self-tuning estimation problems have been studied for systems with unknown parameters and/or noise variances in the past decade [25,26,27,28]. In this paper, only the attack rates and noise variances of the stochastic deception attack signals are unknown. If the model parameters and noise variances of systems are also unknown, the recursive extended least-squares and correlation function can be employed for the identification of unknown model parameters and variances of multiplicative noises, additive noises, and stochastic deception attack signals. This may be more complex, and will be further investigated in future studies.*

### 4.2. DSTFCD

According to the DOFCD obtained by Theorem 1 in Section 3.2 and identified results of the unknown attack rates and noise variances of the deception attack signals in Section 4.1, we can obtain the following distributed self-tuning filtering algorithm based on compressed data.

**Theorem** **2.** *For systems (6) and (22) with the unknown attack rates and noise variances of deception attack signals, the DSTFCD is calculated as*

(40) $${\hat{\bar{x}}}_{i}^{\left(c\right)}\left(t+1|t+1\right)={\hat{\bar{x}}}_{i}^{\left(c\right)}\left(t+1|t\right)+{\hat{K}}_{i}^{\left(c\right)}\left(t+1\right)\left[{\hat{Y}}_{i}^{\left(c\right)}\left(t+1\right)-{\hat{C}}_{i}^{\left(c\right)}\left(t\right){\hat{\bar{x}}}_{i}^{\left(c\right)}\left(t+1|t\right)\right]$$

(41) $${\hat{\bar{x}}}_{i}^{\left(c\right)}\left(t+1|t\right)=A{\hat{\bar{x}}}_{i}^{\left(c\right)}\left(t|t\right)$$

(42) $${\hat{K}}_{fi}^{\left(c\right)}\left(t+1\right)={\hat{P}}_{i}^{\left(c\right)}\left(t+1|t\right){\hat{C}}_{i}^{(c)T}\left(t\right){\left[{\hat{C}}_{i}^{\left(c\right)}\left(t\right){\hat{P}}_{i}\left(t+1|t\right){\hat{C}}_{i}^{(c)T}\left(t\right)+{\hat{Q}}_{V_{i}^{\left(c\right)}}\left(t+1\right)\right]}^{-1}$$

(43) $${\hat{P}}_{i}^{\left(c\right)}\left(t+1|t\right)=A{\hat{P}}_{i}^{\left(c\right)}\left(t|t\right)A^{T}+Q_{\underline{\omega }}\left(t\right)$$

(44) $${\hat{P}}_{i}^{\left(c\right)}\left(t+1|t+1\right)=\left[I_{n}-{\hat{K}}_{i}^{\left(c\right)}\left(t+1\right){\hat{C}}_{i}^{\left(c\right)}\left(t\right)\right]{\hat{P}}_{i}^{\left(c\right)}\left(t+1|t\right),$$

*where ${\hat{\bar{x}}}_{i}^{\left(c\right)}\left(t+1|t+1\right)$ is the self-tuning filter of sensor node i, ${\hat{\bar{x}}}_{i}^{\left(c\right)}\left(t+1|t\right)$ is the self-tuning predictor, ${\hat{K}}_{fi}^{\left(c\right)}\left(t+1\right)$ is the self-tuning filtering gain, and ${\hat{P}}_{i}^{\left(c\right)}\left(t+1|t\right)$ and ${\hat{P}}_{i}^{\left(c\right)}\left(t+1|t+1\right)$ are the corresponding self-tuning prediction error variance matrix and filtering error variance matrix, respectively. The initial values are ${\hat{\bar{x}}}_{i}^{\left(c\right)}\left(0|0\right)=\mu_{0}$ and ${\hat{P}}_{i}^{\left(c\right)}\left(0|0\right)=P_{0}$.*

**Proof.**  By substituting the identified attack rates ${\hat{\bar{\gamma }}}_{i_{k}}(t)$ and noise variances ${\hat{Q}}_{\sigma_{i_{k}}}(t)$ of the stochastic deception attack signals into the distributed optimal filtering algorithm (24)–(28) in Theorem 1, we obtain (40)–(44). This proof is completed. □

The operation of DSTFCD has been summarized in Algorithm 1.
**Algorithm 1:** The DSTFCD algorithm.**Initialization:**Set the initial value at each sensor node *i* with ${\hat{\bar{x}}}_{i}^{\left(c\right)}\left(0|0\right)=\mu_{0}$, ${\hat{P}}_{i}^{\left(c\right)}\left(0|0\right)=P_{0}$, t=0.Step 1: At each sensor node *i*, the measurement data of the neighbor node ${\bar{y}}_{i_{k}}\left(t\right)$ areobtained by
(8).Step 2: Use (30) and (31) to calculate the correlation function $R_{i_{k}}\left(t,0\right)$, $R_{i_{k}}\left(t,1\right)$, use (32)and (33)
to calculate the online identified results ${\hat{\bar{\gamma }}}_{i_{k}}\left(t\right)$, ${\hat{Q}}_{\sigma_{i_{k}}}(t)$.Step 3: Use the identified estimates ${\hat{\bar{\gamma }}}_{i_{k}}(t)$, ${\hat{Q}}_{\sigma_{i_{k}}}(t)$ to calculate the compressedmeasurement
${\hat{Y}}_{i}^{\left(c\right)}\left(t\right)$.Step 4: Substitute the identified estimates ${\hat{\bar{\gamma }}}_{i_{k}}\left(t\right)$, ${\hat{Q}}_{\sigma_{i_{k}}}(t)$ at each time intoEquations (40)–(44)
in Theorem 2. The DSTFCD Algorithm can be obtained.Step 5: Set t=t+1, return to step 1.

### 4.3. Convergence of the Distributed Self-Tuning Filter

**Lemma** **1** ([35])**.** *Consider the following equation,*

(45) δ(t)=F(t)δ(t−1)+u(t),

*where $\delta \left(t\right)\in R^{n}$, $u\left(t\right)\in R^{n}$. F(t) is uniformly asymptotically stable; i.e., there exist constants 0<p<1 and c>0 such that*

(46) $$∥F(t,j)∥\leq cp_{}^{t-j},\forall t\geq j\geq 0,$$

*where F(t,j)=F(t)F(t−1)…F(j+1), $F\left(t,t\right)=I_{n}$. If the input u(t) is bounded, δ(t) is bounded; furthermore, if u(t)→0 as t→∞, δ(t)→0 as t→∞.*

**Lemma** **2** ([35])**.** *Suppose that the n×n matrix Δ(t) satisfies the Lyapunov equation*

(47) $$\Delta \left(t\right)=F_{1}\left(t\right)\Delta \left(t-1\right)F_{2}^{T}\left(t\right)+U\left(t\right),$$

*where U(t) is an n×n input matrix and $F_{1}\left(t\right)$ and $F_{2}\left(t\right)$ are uniformly asymptotically stable matrices. If U(t) is bounded, Δ(t) is bounded; furthermore, if U(t)→0 as t→∞, Δ(t)→0 as t→∞.*

**Assumption** **4.** *Systems (6) and (22) are uniformly completely controllable and observable.*

Based on the DOFCD and DSTFCD, we obtain the following result.

**Theorem** **3.** *The distributed self-tuning prediction and filtering error variances at each sensor node converge to the distributed optimal prediction and filtering error variances with a probability of 1(w.p.1) under any initial values, i.e.,*

(48) $$\lim_{t\to \infty }\left({\hat{P}}_{i}^{\left(c\right)}\left(t+1|t\right)-P_{i}^{\left(c\right)}\left(t+1|t\right)\right)=0,w.p.1$$

(49) $$\lim_{t\to \infty }\left({\hat{P}}_{i}^{\left(c\right)}\left(t|t\right)-P_{i}^{\left(c\right)}\left(t|t\right)\right)=0,w.p.1.$$

**Proof.**  See Appendix A. □

**Theorem** **4.** *The distributed self-tuning predictor and filter at each sensor node converge to the corresponding distributed optimal predictor and filter under any initial values, i.e.,*

(50) $$\lim_{t\to \infty }\left({\hat{\bar{x}}}_{i}^{\left(c\right)}\left(t+1|t\right)-{\hat{x}}_{i}^{\left(c\right)}\left(t+1|t\right)\right)=0,w.p.1$$

(51) $$\lim_{t\to \infty }\left({\hat{\bar{x}}}_{i}^{\left(c\right)}\left(t|t\right)-{\hat{x}}_{i}^{\left(c\right)}\left(t|t\right)\right)=0,w.p.1.$$

**Proof.**  See Appendix B. □

## 5. Simulation Example

**Figure 1:**
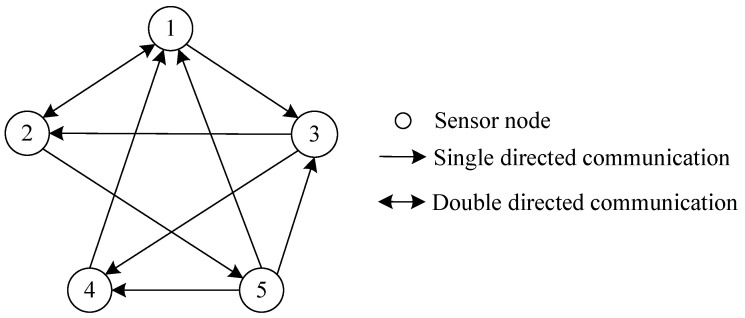
Topology structure of sensor network with five sensors. (authors’ own processing).

To verify the effectiveness and applicability of our algorithms, we consider a target tracking system in practical background consisting of five nodes as an example, the system is made up of mixed uncertainties of multiplicative noises, additive noises, and stochastic deception attacks, whereas the communication between nodes may be under network attack, the sensor network topology structure is given in Figure 1.

The discrete-time system is given as follows:

(52) $$x\left(t+1\right)=\left(A+\sum_{l=1}^{2}\alpha_{l}\left(t\right)A_{l}\right)x\left(t\right)+\left(B+\sum_{l=1}^{2}\beta_{l}\left(t\right)B_{l}\right)\omega \left(t\right)$$

(53) $$y_{i}\left(t\right)=\left(C_{i}+\sum_{l=1}^{2}h_{il}\left(t\right)C_{il}\right)x\left(t\right)+v_{i}\left(t\right),\;\;\;\;\;\;\;i=1,2,\ldots ,5,$$

where the state is $x\left(t\right)=\left[\begin{array}{c} x_{1}\left(t\right)\\ x_{2}\left(t\right) \end{array}\right]$, $x_{1}\left(t\right)$ and $x_{2}\left(t\right)$ respectively denote the position and velocity of the target. $y_{i}\left(t\right)$ is the measurement of the *i*th sensor node. In the simulation, $A=\left[\begin{array}{cc} 0.95 & 0.01\\ 0 & 0.95 \end{array}\right]$, $A_{1}=\left[\begin{array}{cc} 0.1 & 0\\ 0 & 0.01 \end{array}\right]$, $A_{2}=\left[\begin{array}{cc} 0.2 & 0\\ 0 & 0.02 \end{array}\right]$, $B=\left[\begin{array}{c} 0.8\\ 0.6 \end{array}\right]$, $B_{1}=\left[\begin{array}{c} 1\\ 0 \end{array}\right]$, $B_{2}=\left[\begin{array}{c} 0\\ 1 \end{array}\right]$, $C_{1}=\left[\begin{array}{cc} 1 & 0.5 \end{array}\right]$, $C_{2}=\left[\begin{array}{cc} 0.9 & 1 \end{array}\right]$, $C_{3}=\left[\begin{array}{cc} 1 & 1 \end{array}\right]$, $C_{4}=\left[\begin{array}{cc} 1 & 1 \end{array}\right]$, $C_{5}=\left[\begin{array}{cc} 1 & 1 \end{array}\right]$, $C_{i1}=\left[\begin{array}{cc} 1 & 0 \end{array}\right]$, and $C_{i2}=\left[\begin{array}{cc} 0 & 1 \end{array}\right]$, i=1,2,…,5. The process noise ω(t) and measurement noises $v_{i}\left(t\right)$, i=1,2,…,5 are uncorrelated white noises satisfying the relation $Q_{\omega }=0.5$, $Q_{v_{1}}=1$, $Q_{v_{2}}=1$, $Q_{v_{3}}=1$, $Q_{v_{4}}=1$, $Q_{v_{5}}=1$. Variances of multiplicative noises $\alpha_{1}\left(t\right)$, $\alpha_{2}\left(t\right)$, $\beta_{1}\left(t\right)$, $\beta_{2}\left(t\right)$, $h_{i1}\left(t\right)$, and $h_{i2}\left(t\right)$, i=1,2,…,5 are set as $Q_{\alpha_{1}}=Q_{\alpha_{2}}=0.16$, $Q_{\beta_{1}}=Q_{\beta_{2}}=0.11$, $Q_{h_{i1}}=0.21$, and $Q_{h_{i12}}=0.14$. The variances of the random disturbance noises injected into the attack signals are set as $Q_{\sigma_{1}}=9$, $Q_{\sigma_{2}}=3.6$, $Q_{\sigma_{3}}=16$, $Q_{\sigma_{4}}=12$, and $Q_{\sigma_{5}}=4$. The distributions of Bernoulli random variables in attack signals $\gamma_{i}\left(t\right)$, i=1,…,5 satisfy ${\bar{\gamma }}_{1}=E\left\{\gamma_{1}\left(t\right)=1\right\}=0.2$, ${\bar{\gamma }}_{2}=E\left\{\gamma_{2}\left(t\right)=1\right\}=0.4$, ${\bar{\gamma }}_{3}=E\left\{\gamma_{3}\left(t\right)=1\right\}=0.6$, ${\bar{\gamma }}_{4}=E\left\{\gamma_{4}\left(t\right)=1\right\}=0.8$, ${\bar{\gamma }}_{5}=E\left\{\gamma_{5}\left(t\right)=1\right\}=1$. Set the initial values $\mu_{0}=0$ and $P_{0}=I_{2}$.

In this example, our aim is to design DOFCD when the attack rates and noise variances of the stochastic deception attack signals are known, and the DSTFCD when the attack rates and noise variances of the stochastic deception attack signals are unknown.

The performance of DOFCD is depicted in Section 5.1, and the performance of DSTFCD is depicted in Section 5.2.

### 5.1. The Performance of DOFCD

**Figure 2:**
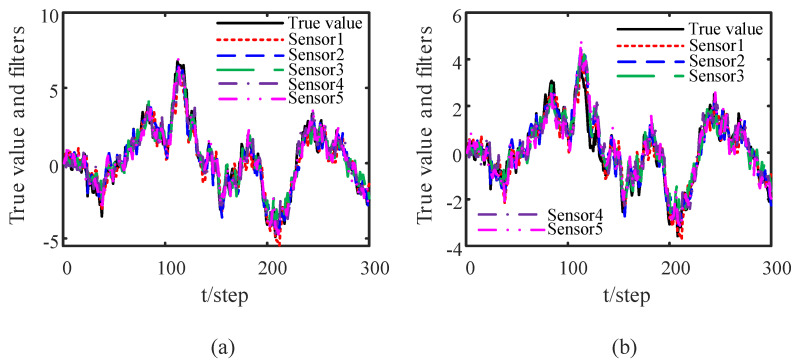
Tracking effects of DOFCDs of five sensor nodes: (**a**) tracking for the position; (**b**) tracking for the velocity (authors’ own processing).

We simulate 300 Monte Carlo runs. Figure 2 shows the tracking effect of the DOFCD in this paper when the attack rates and noise variances of attack signals are known. From Figure 2, we see that the DOFCD has good tracking accuracy.

**Figure 3:**
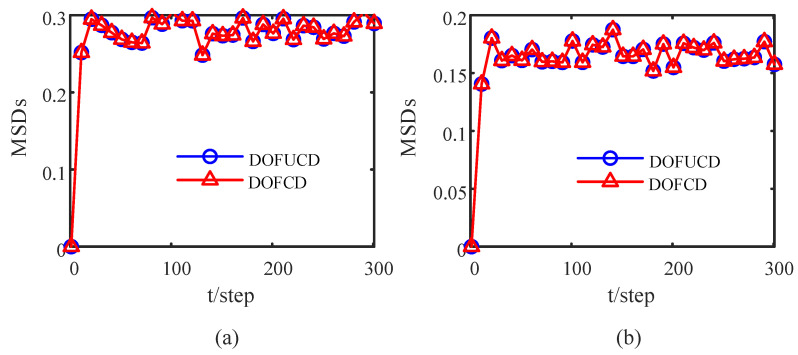
Comparison of MSDs of DOFCD and DOFUCD: (**a**) MSDs of the position filters; (**b**) MSDs of the velocity filters (authors’ own processing).

In this example, the estimation accuracy is evaluated by MSE and MSD, which are defined as

$$\mathrm{MSE}\left(t\right)=\frac{1}{N}\sum_{k=1}^{N}{\left(x^{\left(k\right)}\left(t\right)-{{\hat{x}}_{i}}^{\left(k\right)}\left(t|t\right)\right)}^{2},$$

$$\mathrm{MSD}\left(t\right)=\frac{1}{L}\sum_{i=1}^{L}\left(\frac{1}{N}\sum_{k=1}^{N}{\left(x^{\left(k\right)}\left(t\right)-{{\hat{x}}_{i}}^{\left(k\right)}\left(t|t\right)\right)}^{2}\right),$$

where *N* is the number of Monte Carlo tests. The MSDs of the DOFCD in this paper and DOFUCD in most of the literature are compared in Figure 3. The accuracy of the DOFCD is the same as that of the DOFUCD. However, compared with DOFUCD in most of the literature, the proposed DOFCD has less computational burden than the DOFUCD from Remark 2.

**Figure 4:**
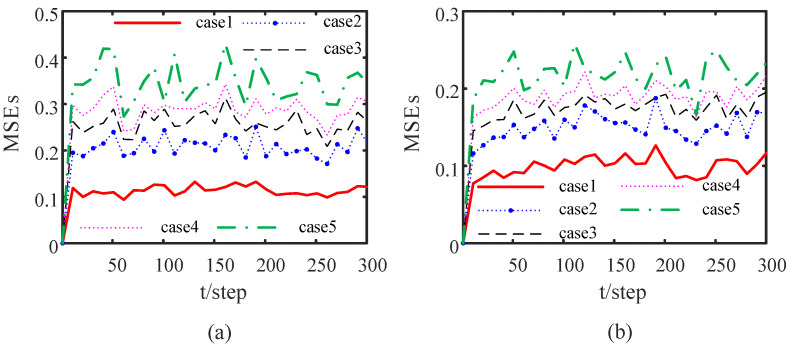
Comparison of MSEs of DOFCDs under different attack rates of the attack signals: (**a**) MSEs of the position filters; (**b**) MSEs of the velocity filters (authors’ own processing).

We consider node 1 as an example. Under the different attack rates of attack signals, the impact of the attack signals on the performance of the DOFCD is shown in Figure 4. The probability distributions of the Bernoulli variables $\gamma_{i}\left(t\right)$, i=2,4,5 of the attack signals injected to the neighbor nodes of sensor 1 are expressed by five cases as follows:

Case 1: ${\bar{\gamma }}_{2}=0$, ${\bar{\gamma }}_{4}=0$, ${\bar{\gamma }}_{5}=0$;

Case 2: ${\bar{\gamma }}_{2}=0.2$, ${\bar{\gamma }}_{4}=0.2$, ${\bar{\gamma }}_{5}=0.2$;

Case 3: ${\bar{\gamma }}_{2}=0.5$, ${\bar{\gamma }}_{4}=0.5$, ${\bar{\gamma }}_{5}=0.5$;

Case 4: ${\bar{\gamma }}_{2}=0.8$, ${\bar{\gamma }}_{4}=0.8$, ${\bar{\gamma }}_{5}=0.8$;

Case 5: ${\bar{\gamma }}_{2}=1$, ${\bar{\gamma }}_{4}=1$, ${\bar{\gamma }}_{5}=1$.

Figure 4 shows that the MSEs of the DOFCDs increase with the increase in the mean ${\bar{\gamma }}_{i}$ of the Bernoulli variables $\gamma_{i}\left(t\right)$, i=2,4,5; i.e., the accuracy of the DOFCD in Case 1 outperforms that in Case 2, that in Case 2 outperforms that in Case 3, that in Case 3 outperforms that in Case 4, and that in Case 4 outperforms that in Case 5. Thus, the greater the attack probability of the attack signal corresponds to a worse accuracy of the DOFCD, which is reasonable.

### 5.2. The Performance of DSTFCD

**Figure 5:**
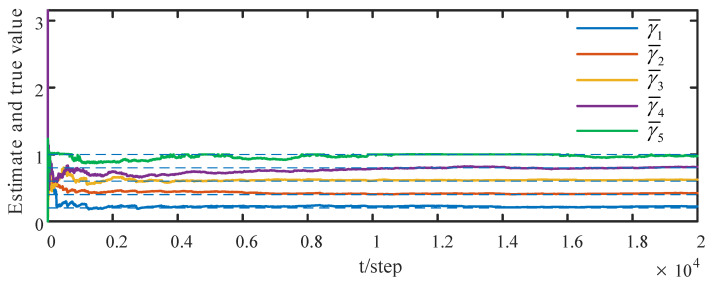
Identified results of unknown attack rates of deception attack signals (authors’ own processing).

**Figure 6:**
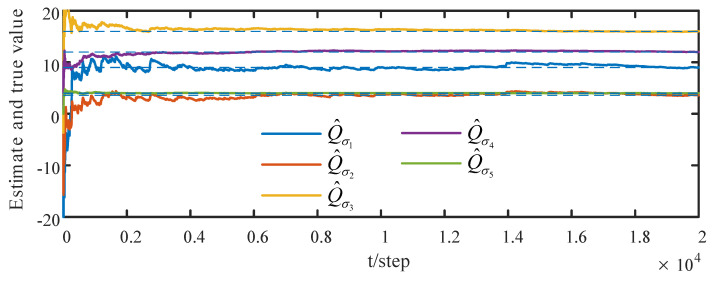
Identified results of unknown noise variances of deception attack signals (authors’ own processing).

**Figure 7:**
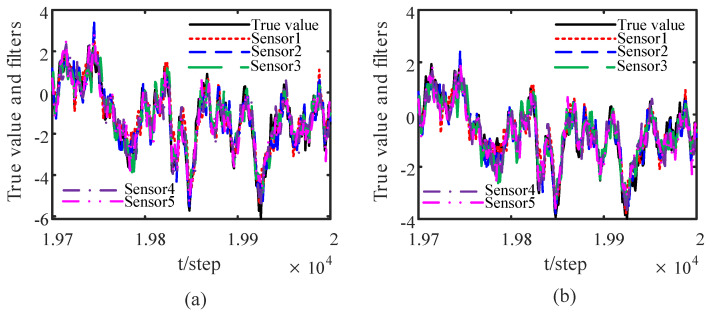
Tracking effects of DSTFCDs of five sensor nodes: (**a**) tracking for the position; (**b**) tracking for the velocity (authors’ own processing).

**Figure 8:**
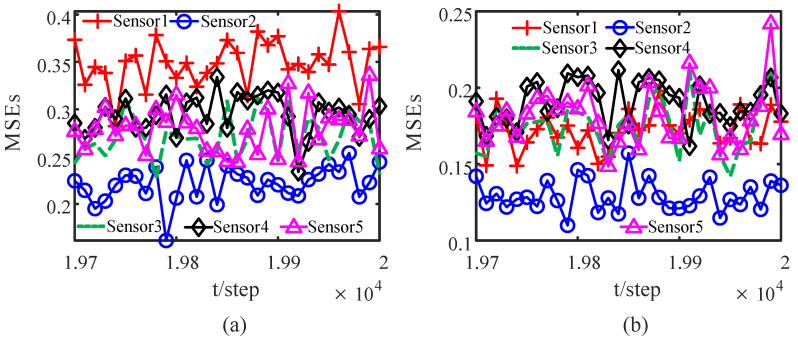
Comparison of MSEs of DSTFCDs of five sensor nodes: (**a**) MSEs of the position filters; (**b**) MSEs of the velocity filters (authors’ own processing).

**Figure 9:**
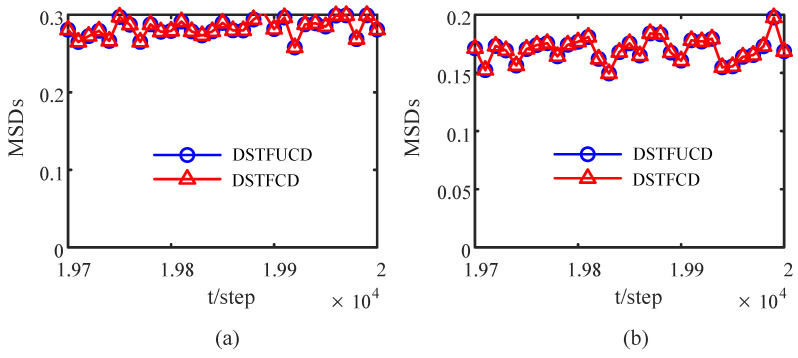
Comparison of MSDs of DSTFCDs and DSTFUCDs of five sensor nodes: (**a**) MSDs of the position filters; (**b**) MSDs of the velocity filters (authors’ own processing).

When the attack rates and noise variances of the deception attack signals are unknown, based on the measurement data of the neighbors of the *i*th sensor node, from (32) and (33) by the correlation function method, we can obtain the identified ${\hat{\bar{\gamma }}}_{i_{k}}\left(t\right)$ and ${\hat{Q}}_{\sigma_{i_{k}}}(t)$. The identified results are provided in Figure 5 and Figure 6. The identified attack rates and noise variances of the attack signals are consistent. That means that the estimates of the attack rates and noise variances converge to their true values as time increases, i.e., (38) and (39) hold. By using the identified results, the tracking effects of the DSTFCDs of five sensor nodes are shown in Figure 7. Figure 8 compares the MSEs of the DSTFCDs for five sensor nodes. From Figure 7 and Figure 8, the DSTFCDs of all nodes have an effective estimation performance. Figure 9 shows the comparison of MSDs of the DSTFCDs and DSTFUCDs for five sensor nodes. From Figure 9, we can see that the DSTFCDs and DSTFUCDs have the same accuracy. Moreover, comparing Figure 3 and Figure 9, the results in Theorem 3 and Theorem 4 can be verified.

**Figure 10:**
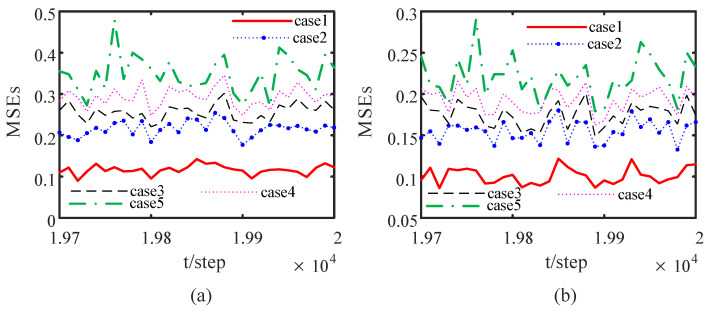
Comparison of MSEs of DSTFCDs with different attack rates of the attack signals: (**a**) MSEs of the position filters; (**b**) MSEs of the velocity filters (authors’ own processing).

Under the same probability distributions of the Bernoulli variables of the attack signals injected to the neighbour nodes of sensor 1 as those in the above DOFCD, Figure 10 shows the effect of the attack rates of the attack signals on the performance of the DSTFCD. A result consistent with Figure 4 is obtained. All simulation results verify the effectiveness of the proposed algorithms.

## 6. Conclusions

For multisensor networked stochastic uncertain systems with multiplicative noise, the measurement data may be attacked by deception attack signals in the process of data exchange between sensor nodes. When the attack rates and noise variances of the attack signal are known, the received augmented high-dimensional measurement is first compressed to a low-dimensional measurement based on the weighted least-squares algorithm. Based on the compressed data, a distributed optimal filter in the LMV criterion was achieved, which had the same accuracy and reduced computational burden compared to that based on uncompressed data. Furthermore, a distributed self-tuning filter based on compressed data was designed when the attack rates and noise variances of the attack signals are unknown, where the correlation function method is adopted to identify the unknown attack rates and noise variances. The convergence of the distributed self-tuning filtering algorithm was analyzed.

In future studies, the distributed security estimation problems will be analyzed for networked stochastic uncertain systems with stochastic deception attacks when model parameters and/or noise covariance in systems are unknown. In addition, the systems may be time-varying and/or nonlinear in practical engineering applications, so the security estimation problems for time-varying systems and nonlinear systems with network attacks will be investigated. Moreover, we will investigate practical applications in target tracking and autonomous navigation in smart vehicles.

## Data Availability

Computer code could be available on request.

## Abbreviations

The following abbreviations are used in this manuscript:

LMV	Linear minimum variance
DoS	Denial-of-service
MSEs	Mean square errors
MSDs	Mean square deviations
DOFCD	Distributed optimal filter based on compressed data
DOFUCD	Distributed optimal filter based on uncompressed data
DSTFCD	Distributed self-tuning filter based on compressed data
DSTFUCD	Distributed self-tuning filter based on uncompressed data

## Appendix A. The Proof of Theorem 3

When the attack rates ${\bar{\gamma }}_{i_{k}}$ and noise variances $Q_{\sigma_{i_{k}}}$, $i_{k}\in N_{i}$ of the stochastic deception attack signals are known, the prediction error covariance of the DOFCD in Theorem 1 satisfies the following optimal time-varying Riccati equation:

(A1) $$P_{i}^{\left(c\right)}\left(t+1|t\right)=A\left[I-K_{i}^{\left(c\right)}\left(t\right)C_{i}^{\left(c\right)}\right]P_{i}^{\left(c\right)}\left(t|t-1\right)A^{T}+Q_{\underline{\omega }}\left(t\right).$$

When the attack rates ${\bar{\gamma }}_{i_{k}}$ and the variances $Q_{\sigma_{i_{k}}}$ of the deception attack signals are unknown, we replace ${\bar{\gamma }}_{i_{k}}$, $Q_{\sigma_{i_{k}}}$ in (16), (22), and (23) by ${\hat{\bar{\gamma }}}_{i_{k}}\left(t\right)$, ${\hat{Q}}_{\sigma_{i_{k}}}(t)$. According to the consistency of identification, i.e., ${\hat{\bar{\gamma }}}_{i_{k}}(t)\to {\bar{\gamma }}_{i_{k}}$, ${\hat{Q}}_{\sigma_{i_{k}}}(t)\to Q_{\sigma_{i_{k}}}$, the following consistency estimates can be obtained:

(A2) $${\hat{Y}}_{i}^{\left(c\right)}\left(t\right)\to Y_{i}^{\left(c\right)}\left(t\right),{\hat{Q}}_{V_{i}^{\left(c\right)}}\left(t\right)\to Q_{V_{i}^{\left(c\right)}}\left(t\right),t\to \infty ,w.p.1.$$

The substitution of ${\hat{Q}}_{V_{i}^{\left(c\right)}}\left(t\right)$ into (A1) yields the distributed self-tuning Riccati equation:

(A3) $${\hat{P}}_{i}^{\left(c\right)}\left(t+1|t\right)=A\left[I_{n}-{\hat{K}}_{i}^{\left(c\right)}\left(t\right)C_{i}^{\left(c\right)}\right]{\hat{P}}_{i}^{\left(c\right)}\left(t|t-1\right)A^{T}+Q_{\underline{\omega }}\left(t\right).$$

The subtraction of (A1) from (A3) yields

$${\hat{P}}_{i}^{\left(c\right)}\left(t+1|t\right)-P_{i}^{\left(c\right)}\left(t+1|t\right)=A\left[I_{n}-{\hat{K}}_{i}^{\left(c\right)}\left(t\right)C_{i}^{\left(c\right)}\right]{\hat{P}}_{i}^{\left(c\right)}\left(t|t-1\right)A^{T}$$

(A4) $$\;-A\left[I-K_{i}^{\left(c\right)}\left(t\right)C_{i}^{\left(c\right)}\right]P_{i}^{\left(c\right)}\left(t|t-1\right)A^{T}.\;\;$$

Let $\Delta {\hat{P}}_{i}^{\left(c\right)}\left(t+1|t\right)={\hat{P}}_{i}^{\left(c\right)}\left(t+1|t\right)-P_{i}^{\left(c\right)}\left(t+1|t\right)$, and $\Delta {\hat{Q}}_{V_{i}^{\left(c\right)}}\left(t\right)={\hat{Q}}_{V_{i}^{\left(c\right)}}\left(t\right)-Q_{V_{i}^{\left(c\right)}}\left(t\right)$, according to (A2), and $\Delta {\hat{Q}}_{V_{i}^{\left(c\right)}}\left(t\right)\to 0$. Equation (A4) is further derived as follows:

$$\Delta {\hat{P}}_{i}^{\left(c\right)}\left(t+1|t\right)=A\left[I_{n}-{\hat{K}}_{i}^{\left(c\right)}\left(t\right)C_{i}^{\left(c\right)}\right]{\hat{P}}_{i}^{\left(c\right)}\left(t|t-1\right)A^{T}-AP_{i}^{\left(c\right)}\left(t|t-1\right){\left[I_{n}-K_{i}^{\left(c\right)}\left(t\right)C_{i}^{\left(c\right)}\right]}^{T}A^{T}$$

$$=A\left[I_{n}-{\hat{K}}_{i}^{\left(c\right)}\left(t\right)C_{i}^{\left(c\right)}\right]\left[{\hat{P}}_{i}^{\left(c\right)}\left(t|t-1\right)-P_{i}^{\left(c\right)}\left(t|t-1\right)\right]{\left[I_{n}-K_{i}^{\left(c\right)}\left(t\right)C_{i}^{\left(c\right)}\right]}^{T}A^{T}$$

$$+A\left[I_{n}-{\hat{K}}_{i}^{\left(c\right)}\left(t\right)C_{i}^{\left(c\right)}\right]{\hat{P}}_{i}^{\left(c\right)}\left(t|t-1\right)C{}_{i}^{(c)T}{\left(K_{i}^{\left(c\right)}\left(t\right)\right)}^{T}A^{T}$$

$$-A{\hat{K}}_{i}^{\left(c\right)}\left(t\right)C_{i}^{\left(c\right)}P_{i}^{\left(c\right)}\left(t|t-1\right){\left[I_{n}-K_{i}^{\left(c\right)}\left(t\right)C_{i}^{\left(c\right)}\right]}^{T}A^{T}$$

$$={\hat{\Psi }}_{i}^{\left(c\right)}\left(t\right)\Delta {\hat{P}}_{i}^{\left(c\right)}\left(t|t-1\right){\left(\Psi_{i}^{\left(c\right)}\left(t\right)\right)}^{T}$$

$$+A{\hat{K}}_{i}^{\left(c\right)}\left(t\right){\hat{Q}}_{V_{i}^{\left(c\right)}}\left(t\right){\left(K_{i}^{\left(c\right)}\left(t\right)\right)}^{T}A^{T}-A{\hat{K}}_{i}^{\left(c\right)}\left(t\right)Q_{V_{i}^{\left(c\right)}}\left(t\right){\left(K_{i}^{\left(c\right)}\left(t\right)\right)}^{T}A^{T}$$

$$={\hat{\Psi }}_{i}^{\left(c\right)}\left(t\right)\Delta {\hat{P}}_{i}^{\left(c\right)}\left(t|t-1\right){\left(\Psi_{i}^{\left(c\right)}\left(t\right)\right)}^{T}+A{\hat{K}}_{i}^{\left(c\right)}\left(t\right)\Delta {\hat{Q}}_{V_{i}^{\left(c\right)}}\left(t\right){\left(K_{i}^{\left(c\right)}\left(t\right)\right)}^{T}A^{T}$$

(A5) $$={\hat{\Psi }}_{i}^{\left(c\right)}\left(t\right)\Delta {\hat{P}}_{i}^{\left(c\right)}\left(t|t-1\right){\left(\Psi_{i}^{\left(c\right)}\left(t\right)\right)}^{T}+U_{i}\left(t\right),$$

where $\left[I-K_{i}^{\left(c\right)}\left(t\right)C_{i}^{\left(c\right)}\right]P_{i}^{\left(c\right)}\left(t|t-1\right)=P_{i}^{\left(c\right)}\left(t|t-1\right){\left[I_{n}-K_{i}^{\left(c\right)}\left(t\right)C_{i}^{\left(c\right)}\right]}^{T}$ has been used in the first equality, $\left[I_{n}-{\hat{K}}_{i}^{\left(c\right)}\left(t\right)C_{i}^{\left(c\right)}\right]{\hat{P}}_{i}^{\left(c\right)}\left(t|t-1\right)C{}_{i}^{(c)T}={\hat{K}}_{i}^{\left(c\right)}\left(t\right){\hat{Q}}_{V_{i}^{\left(c\right)}}\left(t\right)$ has been used in the third equality, $\Psi_{i}^{\left(c\right)}\left(t\right)=A\left[I_{n}-K_{i}^{\left(c\right)}\left(t\right)C_{i}^{\left(c\right)}\right]$, ${\hat{\Psi }}_{i}^{\left(c\right)}\left(t\right)=A\left[I_{n}-{\hat{K}}_{i}^{\left(c\right)}\left(t\right)C_{i}^{\left(c\right)}\right]$, and

(A6) $$U_{i}\left(t\right)=A{\hat{K}}_{i}^{\left(c\right)}\left(t\right)\Delta {\hat{Q}}_{V_{i}^{\left(c\right)}}\left(t\right){\left(K_{i}^{\left(c\right)}\left(t\right)\right)}^{T}A^{T}.$$

According to [35], $\Psi_{i}^{\left(c\right)}\left(t\right)$ and ${\hat{\Psi }}_{i}^{\left(c\right)}\left(t\right)$ are uniformly asymptotically stable matrices under Assumption 4. According to (A2) and (A6), $U_{i}\left(t\right)\to 0,t\to \infty ,w.p.1$. From the uniformly asymptotic stability of $\Psi_{i}^{\left(c\right)}\left(t\right)$ and ${\hat{\Psi }}_{i}^{\left(c\right)}\left(t\right)$ and Lemma 4, it follows that

(A7) $$\Delta {\hat{P}}_{i}^{\left(c\right)}\left(t+1|t\right)\to 0,t\to \infty ,w.p.1.$$

Thus, (48) is true.

Let $\Delta {\hat{P}}_{i}^{\left(c\right)}\left(t|t\right)={\hat{P}}_{i}^{\left(c\right)}\left(t|t\right)-P_{i}^{\left(c\right)}\left(t|t\right)$, similarly, according to the filtering error variances of (28) and (44),

$$\Delta {\hat{P}}_{i}^{\left(c\right)}\left(t|t\right)=\left(I_{n}-{\hat{K}}_{i}^{\left(c\right)}\left(t\right)C_{i}^{\left(c\right)}\right){\hat{P}}_{i}^{\left(c\right)}\left(t|t-1\right)-\left(I_{n}-K_{i}^{\left(c\right)}\left(t\right)C_{i}^{\left(c\right)}\right)P_{i}^{\left(c\right)}\left(t|t-1\right)$$

(A8) $$={\hat{P}}_{i}^{\left(c\right)}\left(t|t-1\right)-P_{i}^{\left(c\right)}\left(t|t-1\right)-{\hat{K}}_{i}^{\left(c\right)}\left(t\right)C_{i}^{\left(c\right)}{\hat{P}}_{i}^{\left(c\right)}\left(t|t-1\right)+K_{i}^{\left(c\right)}\left(t\right)C_{i}^{\left(c\right)}P_{i}^{\left(c\right)}\left(t|t-1\right).$$

Let $\Delta {\hat{K}}_{i}^{\left(c\right)}\left(t\right)={\hat{K}}_{i}^{\left(c\right)}\left(t\right)-K_{i}^{\left(c\right)}\left(t\right)$, (A8) can be rewritten as

$$\Delta {\hat{P}}_{i}^{\left(c\right)}\left(t|t\right)={\hat{P}}_{i}^{\left(c\right)}\left(t|t-1\right)-P_{i}^{\left(c\right)}\left(t|t-1\right)-K_{i}^{\left(c\right)}\left(t\right)C_{i}^{\left(c\right)}{\hat{P}}_{i}^{\left(c\right)}\left(t|t-1\right)$$

$$-\Delta {\hat{K}}_{i}^{\left(c\right)}\left(t\right)C_{i}^{\left(c\right)}{\hat{P}}_{i}^{\left(c\right)}\left(t|t-1\right)+K_{i}^{\left(c\right)}\left(t\right)C_{i}^{\left(c\right)}P_{i}^{\left(c\right)}\left(t|t-1\right)$$

(A9) $$=\left[I_{n}-K_{i}^{\left(c\right)}\left(t\right)C_{i}^{\left(c\right)}\right]\Delta {\hat{P}}_{i}^{\left(c\right)}\left(t|t-1\right)-\Delta {\hat{K}}_{i}^{\left(c\right)}\left(t\right)C_{i}^{\left(c\right)}{\hat{P}}_{i}^{\left(c\right)}\left(t|t-1\right).$$

According to (26), (42), (A2), and (A8), $\Delta {\hat{K}}_{i}^{\left(c\right)}\left(t\right)\to 0,t\to \infty$. Furthermore, according to (A9), $\Delta {\hat{P}}_{i}^{\left(c\right)}\left(t|t\right)\to 0$, i.e., (49) is true. This proof is completed.

## Appendix B. The Proof of Theorem 4

According to Theorems 1 and 3, the distributed optimal and self-tuning predictors is rewritten as

(A10) $${\hat{x}}_{i}^{\left(c\right)}\left(t+1|t\right)=\Psi_{i}^{\left(c\right)}\left(t\right){\hat{x}}_{i}^{\left(c\right)}\left(t|t-1\right)+AK_{i}^{\left(c\right)}\left(t\right)Y_{i}^{\left(c\right)}\left(t\right)$$

(A11) $${\hat{\bar{x}}}_{i}^{\left(c\right)}\left(t+1|t\right)={\hat{\Psi }}_{i}^{\left(c\right)}\left(t\right){\hat{\bar{x}}}_{i}^{\left(c\right)}\left(t|t-1\right)+A{\hat{K}}_{i}^{\left(c\right)}\left(t\right){\hat{Y}}_{i}^{\left(c\right)}\left(t\right).$$

Let $\delta_{i}\left(t\right)={\hat{\bar{x}}}_{i}^{\left(c\right)}\left(t|t-1\right)-{\hat{x}}_{i}^{\left(c\right)}\left(t|t-1\right)$, we can obtain the dynamic error equation

(A12) $$\delta_{i}\left(t+1\right)=\Psi_{i}^{\left(c\right)}\left(t\right)\delta_{i}\left(t\right)+u_{i}\left(t\right)$$

(A13) $$u_{i}\left(t\right)=\Delta {\hat{\Psi }}_{i}^{\left(c\right)}\left(t\right){\hat{\bar{x}}}_{i}^{\left(c\right)}\left(t|t-1\right)+A{\hat{K}}_{i}^{\left(c\right)}\left(t\right){\hat{Y}}_{i}^{\left(c\right)}\left(t\right)-AK_{i}^{\left(c\right)}\left(t\right)Y_{i}^{\left(c\right)}\left(t\right),$$

where $\Delta {\hat{\Psi }}_{i}^{\left(c\right)}\left(t\right)={\hat{\Psi }}_{i}^{\left(c\right)}\left(t\right)-\Psi_{i}^{\left(c\right)}\left(t\right)$. We can easily obtain that $\Delta {\hat{\Psi }}_{i}^{\left(c\right)}\left(t\right)\to 0$. According to the proof of Theorem 4, $u_{i}\left(t\right)\to 0$. According to the uniformly asymptotic stability of $\Psi_{i}^{\left(c\right)}\left(t\right)$ and Lemma 3, $\delta_{i}\left(t\right)\to 0$ as t→∞, which leads to (A10). Similarly, (A11) can be proven. Hence, the distributed self-tuning predictor and filter have asymptotic optimality. This proof is completed.
